# Perceived behavioral problems of school aged children in rural Nepal: a qualitative study

**DOI:** 10.1186/s13034-015-0061-8

**Published:** 2015-06-26

**Authors:** Ramesh P. Adhikari, Nawaraj Upadhaya, Dristy Gurung, Nagendra P. Luitel, Matthew D. Burkey, Brandon A. Kohrt, Mark J.D. Jordans

**Affiliations:** Research Department, Transcultural Psychosocial Organization (TPO), Kathmandu, Nepal; Padma Kanya Multiple Campus, Tribhuvan University, Kathmandu, Nepal; Division of Child and Adolescent Psychiatry, Johns Hopkins School of Medicine, Baltimore, USA; Department of Psychiatry and Behavioral Sciences, Duke Global Health Institute, Duke University, Durham, USA; Research and Development, HealthNet TPO, Amsterdam, The Netherlands; Center for Global Mental Health, Institute of Psychiatry, Psychology and Neuroscience, King’s College London, London, UK

**Keywords:** Child behavior problems, Nepal, Psychosocial, Qualitative

## Abstract

**Background:**

Studies on child behavioral problems from low and middle income countries are scarce, even more so in Nepal. This paper explores parents’, family members’ and teachers’ perceptions of child behavioral problems, strategies used and recommendations to deal with this problem.

**Method:**

In this study, 72 free list interviews and 30 Key Informant Interviews (KII) were conducted with community members of Chitwan district in Nepal.

**Result:**

The result suggest that addictive behavior, not paying attention to studies, getting angry over small issues, fighting back, disobedience, and stealing were the most commonly identified behavioral related problems of children, with these problems seen as interrelated and interdependent. Results indicate that community members view the family, community and school environments as being the causes of child behavioral problems, with serious impacts upon children’s personal growth, family harmony and social cohesion. The strategies reported by parents and teachers to manage child behavioral problems were talking, listening, consoling, advising and physical punishment (used as a last resort).

**Conclusions:**

As perceived by children and other community dwellers, children in rural Nepalese communities have several behavioral related problems. The findings suggest that multi-level community-based interventions targeting peers, parents, teachers and community leaders could be a feasible approach to address the identified problems.

## Introduction

One third of the world’s population are children and adolescents, with the majority living in low and middle-income countries (LMICs) [1] The World Health Organization estimates that neurological, mental and behavioral disorders and self-harm contribute 12 % of the global burden of disease [2]. Half of neuropsychiatric disorders are estimated to have onset by the age of 14 [3]. Globally, around 10 to 20 % of children and adolescents suffer from a mental health problem [1] and suicide is one of the top three leading causes of death among adolescents [4].

Conduct or behavior problems include problems related to repeated violation of other’s rights, aggressiveness, hyperkinetic impulsive behavior, and missing classes or running away from school [5, 6]. The Global Burden of Disease report 2010 indicates that conduct disorder is among the 15 leading causes of disability adjustment life years of children ages 5–19 years [7]. A study conducted in five developing countries suggest that 10.5 % of adolescent suffer from mental health problems, with significant proportion being conduct problems [8]. Likewise, 20.8 % of children in Brazil [9], 11.7-13.7 % of school age children in Sri Lanka [10], 34-36 % of children in Pakistan [11], and 30 % of children in India [12] suffer conduct or behavioral problems.

Despite the importance of gathering evidence on child behavior problems for individual development and public health, there remains lack of data from low and middle-income countries [1, 13]. Studies conducted in developed countries have shown that child conduct or behavior problems have negative impacts on children’s social, educational and economic performance in later life [14, 15]. Childhood behavior problems also predict involvement in anti-social behavior in adulthood [16].

Nepal is one of the least developed countries in the world in term of human development indicators with a Human Development Index (HDI) of 0.54 and a Gender Inequality Index (GII) of 0.48 [17] The most recent 2011 national census counted a total population of 26.3 million; 44.4 % of the Nepali population are children (0–17 years). The majority of the population (83 %) reside in rural areas [18]. In Nepal children suffer socio-economic problems including nutrition, shelter, domestic violence, forced labour, caste/ethnic discrimination and lack of access to basic education and medical treatment [19]. The 10-year long armed conflict between the Government of Nepal and the Communist Party of Nepal (Maoist) has also had a significant impact on children, including death, injury, abduction, displacement, abuse, and the disappearance and killing of family members and relatives [20, 21]. Moreover, many children and adolescents in Nepal suffer from psychosocial and mental health problems related to family break-up, changing family structure, domestic violence, and parental substance abuse [22].

Behavior problems have not been thoroughly assessed among children in Nepal; however, a study of psychosocial counseling in Nepal reported aggression was one of the most common reasons for presentation for mental health care [23]. In another study, compared to other mental health and psychosocial problems, aggression explained the greatest amount of variance in cortisol as a marker of hypothalamic pituitary adrenal axis functioning among Nepali boys [24]. Prior studies [25] have relied upon diagnostic checklists developed for use in high income settings, with unknown validity or reliability in Nepal. Furthermore, existing studies do not address the context in which the reported symptoms occurred, their association with functional problems, or the significance of the symptoms as attributed by family members. Finally, the limited contextual information about the behavior problems from previous reports does little to inform the content or form of potential interventions to address the identified problems.

There is a need for increased research in low-income settings to increase understanding of child behavior problems in order to explore feasible, acceptable, and effective ways of addressing such problems. Qualitative research is best suited for understanding cultural and contextual factors that are affecting mental health of the population [26]. Thus, the current qualitative study aims to assess parents’, family members’ and teachers’ perceptions of children’s behavioral problems, and their ideas to address the identified problems.

## Methods

### Study area

This study was conducted in Jutpani and Meghauli Village Development Committees (VDCs) of Chitwan district in Nepal. Of the total population (Meghauli: 16,252; Jutpani: 15,118) around two fifths are children in each VDC. The majority ethnic/caste groups in the study VDCs are Janjati, followed by Brahmin/Chhetris, and Dalits. This study targeted children aged 8–15 years as well as parents, community members, and school teachers identified as key stakeholders given their direct interactions with children and their potential influence on children’s behavior.

### Design

Two qualitative methods (free list interviews and key informant interviews) were used for data collection. Free list interviews provide a broad overview of a community’s perception of problems [27]. The open ended question “Please tell us about the problems children between 8–15 years are facing in your community” was asked during the free list interviews to identify the general problems of children in the community. Initial data analysis reviewed the list of general problems, identifying and categorizing the behavior or conduct problems that were most frequently mentioned. The five most frequently reported behavior problems were then explored in detail in key informant interviews. Key informant interviews specifically focused on probing related to identification of the problem, its perceived causes, perceived effects, what children/caregivers have done to address these problems, and what else could be done to minimize the problems.

### Sample

Altogether 72 free list interviews and 30 Key Informant Interview (KII) were conducted. The respondents of free list interviews consisted of parents (caregiver) and children, and were selected purposively based on pre-determined criteria of living in the study area, available during the study period and willing to talk with the interviewers. Among the 72 free list respondents, 24 were children aged 8–15 years (12 boys and 12 girls) and 48 were parents of children aged 8–15 years (24 mothers and 24 fathers – not necessarily the parents of the children included in the study). The rationale behind taking two distinct groups of respondents is to capture all the problems related to children in the community. Some child related problems are only observed by children whereas others only by parents. In free-list interview the age of children ranged from 8 to 15 years with mean 12.9 years and parents age ranged from 26 to 55 years with mean 36.4 years.

For the study, key informants were identified as per the recommendation of free list interviews participants, and through consultation with community leaders. The identified key informants were schoolteachers (*n* = 8), community people (*n* = 7), social workers (person involved in community welfare activities) (*n* = 4), Female Community Health Volunteers (FCHVs) (*n* = 4), members of community based organization/ non-governmental organization (*n* = 3), members of youth clubs (*n* = 2), and participants in women’s groups (n = 2). The age of key informants ranged from 20 to 55 years with mean 36.7 years and education ranged from primary level to Master’s degree.

### Procedure

The study was conducted during August to September 2013. One supervisor and four research assistants with several years of experience in mental health research were involved in data collection. The team received one-week training on the research objectives, research methodology and interview processes, and research ethics.

Free list interviews were recorded through a verbatim written transcript in Nepali. All key informant interviews were audio recorded along with note taking, with recordings subsequently transcribed into Nepali for analysis. Relevant results were translated into English for the purpose of analysis, with key Nepali phrases provided in italics in this manuscript. Each of the free list interviews lasted from 30 min to 45 min, while the KIIs lasted from 45 min to 120 min.

### Analysis

Analysis followed the Design, Implementation, Monitoring and Evaluation (DIME) Model procedure for analyzing free list and key informant interview data [28]. The DIME Model states that variations in culture and environment affect how mental health and psychosocial problems are described, understood and prioritized by the local community. For this reason, the DIME model was adopted for the Nepalese context, in particular taking consideration of local socio-cultural aspects of villages in Chitwan district. At first, the free list data were analyzed by the research team by listing all the problems in a single summary table with frequency of each problem calculated based on the number of times the problem was reported by respondents. The main objective of free-list interview was to list all the problems faced by children; therefore, the results of the free-list with children and parents are presented together in the results section.

The summary sheets for the five most reported behavioral problems explored in KIIs was then prepared, which consisted of five subheadings based on the questions and probes: (a) symptoms of the problems; (b) perceived causes; (c) perceived impact; (d) what is currently done to address this problem; and (e) recommendations to address the problem. Responses were listed under the subheadings of each behavioral problem. The frequencies were calculated based on how many respondents reported each item. If more than two respondents mentioned same concepts but in different phrasing, then the research team selected the most appropriate phrase to represent the problem through consensus, and added the frequency.

### Ethical approval

The study received ethical approval from the Nepal Health Research Council (NHRC) (Reg. no. 112/2013). Prior to the interview, all participants were asked for written consent. In case of children, oral assent was obtained from the child and written informed consent was also obtained from the child’s parents. All study participants were informed of their right to refuse participation and to leave the interview at any time.

## Results

### General problems of children

The most common general problem of children in this community reported by the 72 participants of free list were economic problems (*aarthik samasya*) (*N* = 51), not paying attention to (i.e., neglecting) school work (*padhaima dhyan nadine*) (N = 48), involvement in addictive behaviors (*kulat ma fasne*) (*N* = 46), family and households problems (*gharayesi samasya*) (N = 40), getting angry easily and fighting over small issues (*sano kurama risaune/jhagada garne*) (*N* = 31), disobedience (*atteri)* (*N* = 25), and stealing (*chorn*e) (*N* = 20). “Economic problems” included problems related to meeting basic requirements such as nutritious food, clothing, school materials and medicines. “Not paying attention to studies” included dropping out, irregular school attendance, lack of interest in education, not doing homework properly, skipping classes, always failing exams, roaming around during school hours, being more interested in playing than studying, and always watching television instead of studying. Family and household problems included having to spend too much time in household chores (such as cooking food, herding cow, goats etc.); not receiving proper care or supervision from parents as they are busy with work, having many siblings in the household, being orphaned, parents consuming alcohol and having disputes in the households; and not being allowed to participate in community activities.

### Major behavioral problems of children

The five most reported behavior problems identified from free list interviews were (1) not paying attention to studies (*padhaima dhyan nadine*), (2) involvement in addictive behaviors (i.e., consuming alcohol, cigarettes and marijuana) (*kulat ma fasne*), (3) getting angry easily and fighting over small issues (*sano kuramarisaune/jhagadagarne*), (4) disobedience (*atteri*), and (5) stealing (*chorne*). These problems were explored extensively during subsequent key informant interviews, the results of which are presented in Table 1 and in the text below.

**Table 1:** Major behavioral problems of children discussed in 30 key informant interviews

Reported major behavior problem (*N* = number of key Informants)	Signs and Symptoms (*n* = number of frequencies)	Causes (*n* = number of frequencies)	Effects (*n* = number of frequencies)
Addictive behaviors including: drinking alcohol, smoking cigarettes/marijuana (*Kulat ma fasne*)	Speak rudely or use foul language (17)	Unfavorable family environment (illiteracy, bad habits, addiction of parents, family discord) (14)	Family, teachers, friends and community views them negatively/don’t like them or care for them/demean them (17)
(*N* = 17)	Fight with, threaten/beat friends, family, teachers, and community) (17)	Bad influence of friends (13)	Negative effects in their education (expulsion from school, failure in exams, discontinue education) (16)
	Roam around the neighborhood (14)	Parents don’t pay attention to their children (13)	Display other behavioral problems (involvement in criminal activities, become violent towards others) (12)
	Become disobedient (9)	Lack of awareness (of parents and children) (10)	Negative effects in neighbors and community (loss of social cohesion, disturbance of peace) (10)
	Steal other’s possessions (8)	Poverty (9)	Negative effects in the family (family discord, weaken economic condition of family) (9)
	Stay out until midnight (8)	Due to favorable environment (availability of secluded area like forests, parent don’t care, allowances in castes and community) (9)	Their future becomes dark (8)
	Don’t concentrate in their studies/don’t care about their studies (7)	Too much love and trust of parents towards their children (3)	Health gets worse (8)
	Don’t go to school regularly/bunk classes and schools (10)	For fun (3)	Have to bear physical/verbal punishments (6)
	Drop-out of school (6)		Feel humiliated, isolated, stressed and guilt (6)
	Form gangs (6)		Others don’t allow their children to socialize with them/avoid them (4)
	Get angry even on small matters (6)		Have to be involved in income generating activities (4)
	Tell lies (5)		
	Try to look and behave nicely in front of others (3)		
	Become arrogant (3)		
	Wear rough/indecent clothes (3)		
	Don’t care about daily activities (eating, cleaning) (3)		
	Play cards outside (2)		
	Walk around inebriated and reek of alcohol (4)		
Not paying attention to studies (drop-out, irregular school attendance, lack of interest in education) (*Padhai ma dhyan nadine*)	Show problems in school (don’t go to school regularly/bunk school and classes/ don’t pay attention to their studies/don’t do homework/disrupt classroom by making noise) (11)	Scarcity of teachers in the school (11)	Despised, disgraced and mistreated by others (10)
(*N* = 11)	Wander around in the neighborhood at the time of school (7)	Lack of good family environment (parents are busy, parents cannot spare time for children, being orphan) (11)	Get caught up in bad habits (addiction, stealing) (7)
	Get angry/irritated and talk back to others (6)	Weak economic condition and not being able to fulfill the need and demands of children (9)	Be bad influence to youngsters (4)
	Disobey family members and teachers (11)	Spoilt by parents (too much love, fulfilling excessive demands) (8)	Fail in exams/ruin education (5)
	Fight with friends or don’t care about them (6)	Lack of awareness/education of parents (7)	Have to be involved in labor work (4)
	Lie to parents and teachers (4)	Bad influence of friends (6)	Bleak future (3)
	Get caught up in bad habits (4)	Lack of good environment in the community (3)	Feel bad or depressed (3)
	Don’t speak properly with others (3)	Exploitation of communication/technology (2)	Make parents worried (2)
	Not afraid of teachers (2)	Disciplining by teachers (2)	Not stay at home for long (2)
	Donning undesirable fashion (long hair, earring) (2)		
	Vandalizing other’s properties (2)		
	Don’t take care about personal hygiene (2)		
Get angry easily and fight over small issues (*Sano kurama risaune/jhagada garne*)	Become aggressive in small matter (7)	Unfulfilled needs/desire (7)	Hinders their studies (drop out, get expelled from school) (7)
(*N* = 7)	Lack of interest in studies (6)	Carelessness, illiteracy of the family members (6)	Community view them and their family negatively (6)
	Speak rudely/talk back and argue with others (4)	Lack of congenial family environment (discord, addictions, bad behavior of parents) (6)	Increase in emotional problems (feeling anxious, tensed, thinking too much) (4)
	Not listening to other’s advice (3)	Due to bad influence of friends (4)	Increase in aggressive behaviors (4)
	Done undesirable fashion (wear earrings, color hair) (3)	Parents don’t enrolled their children in schools (4)	Have to bear reprimands from parents (3)
	Become involved in addictive behaviors (3)	Because they are teenagers (3)	Have to be involved in lawsuits (2)
	Form groups/gangs (3)	Due to fights between friends (2)	Affect others due to one’s actions (2)
		Emulating others (2)	Discord in the family (2)
		Biological reasons (heredity, hormones) (2)	
		Early marriage (2)	
Disobedience (*Atteri*)	Don’t listen to others (5)	Poor economic condition (7)	Affects their studies (7)
(*N* = 7)	Become aggressive and quarrel with others (5)	Spoilt (due to good economic condition and over indulgence) (4)	No one will like them/view them negatively (6)
	Talk about indecent things (4)	Parents don’t take good care of their children (4)	scolding and beatings by other (3)
	Say whatever comes to mind (4)	Parents beat children after drinking alcohol (3)	Increase in negative thoughts (3)
	Lack of interest in studies/don’t do homework (4)	Children try to emulate others (3)	Don’t focus on their daily activities (2)
	Try to circumvent/make excuses for tasks given to them (4)	Don’t do homework (2)	
	Are arrogant and brag (3)	Heredity (2)	
	Steal (3)		
	Are not afraid of teachers (3)		
	Vandalize other’s properties (2)		
	Mischievous (2)		
	Are disruptive in classroom (2)		
	Get influenced easily (2)		
Stealing (*Chorne*)	Steal (from home and community) (4)	Weak economic condition of family (3)	Could become a criminal (thief)/could be jailed (3)
(*N* = 3)	Argue with parents/become disobedient (4)	Family incapable of convincing them (3)	Parents of friends don’t allow their children to socialize with them (3)
	Bunk school (3)	Peer influence (2)	Are disgraced by their family (2)
	Consume alcohol/cigarette (3)	Parents don’t provide good care to the children (2)	Community view them negatively (2)
	Argue with community members (2)		Have bleak future (2)
	Don’t concentrate on their studies (2)		

“Addictive behaviors” included drinking alcohol, smoking cigarettes or marijuana and using other drugs. This problem was reported to be common among children who used foul language, fought or threatened others, and roamed around the neighborhood aimlessly. Not paying attention included: not going to school regularly, skipping classes, showing less interest in education, wandering around the neighborhood aimlessly. Other behavioral problems identified by the participants included: ‘get angry easily and fight over small issues’, ‘disobedience’ and ‘stealing’.

The major causes of the problems reported by respondents were unfavorable family environment such as domestic violence, alcohol abuse, discrimination between son and daughter etc., negative peer influence, poverty, lack of awareness of community members, lack of attention or too much attention from parents, unfulfilled needs and wants, and lack of a good school environment. These problems were said to result in mistreatment of children by others, have a negative impact in education, lead to involvement in other bad habits and activities (such as addiction, stealing), increase in emotional problems, and have negative impacts on personal and family image (or social status).

Most participants reported that behavioral problems were common among boys, children aged 12–15 years, children from Janajati and Dalit communities, those children who have dropped out of school, and those living in poorer economic conditions.

### Existing practices and suggestions to address the problems

Most participants reported that family members, community people, teachers and friends try to convince children to correct their behavior through verbal discussions or instructions. It was also found that parents and teachers punish children physically if verbal discussions or convincing do not work. Teachers and community people reported visiting children’s homes and talk to their parents about their child’s behavioral problems. Sometimes parents also go to their child’s school and ask teachers to try to correct their child’s behavior by talking to them and by punishment.

The respondents’ main suggestions to address behavior problems in the local community were: to make the children, parents and schoolteacher aware of the problems; to talk with the children and provide suggestions; and to provide individual and family support for children suffering from severe behavioral problems.

## Discussion

This study conducted in rural Nepal identified several child behavioral problems that were concerning to parents, teachers, and other community members. In this section we discuss: 1) the most commonly reported behavioral problems and their inter-relations; 2) the perceived roles of peers, family, and the community environment in shaping child behavioral problems; 3) the impact of behavioral problems on the individual child and those around him or her; and 4) strategies used by parents and teachers to manage child behavioral problems in the study community.

Respondents in our study primarily understood behavioral problems in terms of school performance, addictive behaviors and antisocial behaviors. Many of the problems reported by parents, teachers, and community members related were those that directly affected school attendance and performance. Adults were also highly concerned about children and adolescents using substances, with particular emphasis placed on cigarettes, marijuana, and alcohol. Informants were concerned about behaviors that suggested lack of discipline (e.g., wandering aimlessly, disobeying) or that had direct negative effects on others (e.g., stealing, speaking rudely to others, and being aggressive.

School-related behavioral problems included dropping out, irregular in school attendance, lack of interest in schoolwork and education, not completing homework, skipping class, always failing exams, roaming around during school hours, being more interested in playing than studying and always watching television instead of studying. The addiction related problems included smoking cigarettes or marijuana, and substance misuse (drinking alcohol and using drugs). Antisocial behaviors identified were: wandering around the neighborhood aimlessly, stealing, speaking rudely, threatening others, not listening to others, and becoming aggressive without reason.

The major behavioral problems found in our study significantly overlap with the findings reported by Alisausks and Simkiene [6] in terms of aggression, skipping classes and stealing. However, the findings by Alisausks and Simkiene differ in terms of violent manifestation of problems such as “cruelty with animals or people, deliberate fire-raising, attempts of rape” and use of weapons to harm others [6] were not reported in our study.

The behavioral problems identified in our study are seen in the general child population, but respondents strongly felt that boys have more behavioral problems than girls. This is an agreement with the findings of study conducted by Ojha and colleagues [29] who found the prevalence of emotional and behavioral problems in Nepalese children aged 4–18 was 31.6 % among boys and 22.4 % among girls. This could be attributed to increased externalizing behaviors among boys [30]. In Nepal, this could be explained because there are fewer checks and balances on son’s behavior due to the cultural belief that relies on sons to perform the death rights which opens the gate for heaven [31]. Similarly, different treatment of boys and girls in the family and community could be responsible for the degree of difference in behavioral problems reported. For example, Nepalese child rearing practices and socialization patterns often impose stricter behavioral regulations on girls than on boys [30].

Similar to a study by Atilola and colleagues, [8] participants in our study believed that behavioral problems were more common among older children (i.e., aged 10–14 years), orphans, children from marginalized communities, and poor families. This might be because of an inability to deal with day-to-day stress, lack of social protection and parental guidance [32] and difficulties dealing with physical and psychological changes of childhood. It might also represent a biased perspective among the participants, for example with regards to the finding around marginalized groups.

The most frequently mentioned causes of child behavioral problems are an unfavorable family environment (domestic violence, alcohol abuse, discrimination between son and daughter etc.) and lack of attention or too much attention from parents. Similar findings were reported in a study by Vijayalakshmi and colleagues [30] where child rearing practices such as parent’s supportive interactions with children, providing a stimulating environment, meeting basic needs and proving appropriate leisure activities were associated with a reduction in child behavioral problems. Our study respondents believed that, besides parents’ attitudes and the family environment, other factors such as negative societal attitude and peer influence were equally responsible for child behavioral problems. This is in agreement with findings of other studies [33–37]. Parents and family members are generally chiefly responsible for their child’s development of a sense of morality and their education. In Nepal, some families do not manage to fulfill this role due to livelihood difficulties and lack of available time [38], while others are not managing due to their personal attitude and behavior [39].

A retrospective case control study conducted in Vellore, Tamil Nadu found that child-rearing practices influence children’s behavior [30]. Negative societal attitudes toward children with behavioral problems further exacerbate the problem. As such, children are neglected, socially isolated and often ostracized. Due to unsupportive behavior of community members and lack of societal mechanisms to bring the children back into normal life, some children with behavioral problems go on to become involved in bigger crimes. Furthermore friendship and proximity to peers involved in anti-social behaviors is a potential cause of child behavioral problems.

Respondents in this study believed that behavioral problems result in mistreatment of affected children by others, negative impact on education, involvement in other bad habits and activities (such as addiction, stealing), increase in emotional problems, and negative effects in personal and family image. This is consistent with the findings of Engle and colleagues [40] who highlighted the negative impact of behavioral problems on children’s education. Other studies suggest that children with behavioral problems are more likely to be involved in anti-social behavior in adulthood [16], and that there are socio-economic disadvantages for the families of children with behavioral problems [41].

Parents and teachers in our study reported have difficulties managing children’s behavioral problems. Currently, there are no programs from governmental or civil society organizations addressing child behavioral problems in the study community. Parents, peers and teachers try to manage child behavioral problems by talking to the child, providing moral education and instructions. When this does not work, parents and teachers also physically punish the child. “Beating for betterment” [42] is a commonly recognized concept in Nepal and continues to be practiced in communities like the one evaluated in this study.

### Implications

Based on the findings of the study child behavioral problems should be addressed at several levels including family, school and community. For this, a multi-level intervention is needed. At the family level, a parenting intervention addressing healthy child rearing practices may help address discipline practices and monitoring. At the community level, awareness programs on the impact of behavioral problems on children, family and communities can be run with the help of community radio, televisions and mobile phones [40]. The development of information, education and communication (IEC) materials on behavioral problems and their dissemination among children, parents and teachers could help in the management of behavioral problems. These materials could be discussed in a parent-teacher group discussion program [43] to strengthen the combined teacher-parent efforts to deal with child behavioral problems.

### Limitations

This study was conducted in relatively small geographical area, and by selecting respondents purposively; therefore, the results may not be representative for other settings in Nepal. Second, the one-time free-list interviews and semi-structured interviews might not have captured all forms of behavioral problems. As most behavioral problems are better noticed through regular observation of behaviors and practices within the family and community, an ethnographic method may offer additional insights. However, time and resource limitations prevented this; instead, a qualitative design employing open-ended questioning to capture respondents’ perspectives was selected to balance the goals of representativeness, depth, and efficiency [28]. Another limitation could be that the wide age range (8–15 years) might have affected the responses of children in which children 8–12 provided less information compared with 13–15 years of age.

## Conclusion

The most commonly reported child behavioral problems in the study area were addictive behaviors, neglecting schoolwork, getting angry over small issues, fighting, disobedience, and stealing. The children’s family, school, and community environments were seen responsible for the increase or decrease of these problems. Children with supportive parents and family environments were thought to exhibit fewer behavior-related problems than children with unsupportive parents and difficult family circumstances. Respondents reported that society had negative attitudes towards children with behavioral problems, with almost no systems or mechanisms in place to address such problems. Parents and teachers have difficulties managing children’s behavioral problems. Parents and teachers reported used listening, talking and consoling as a first option to deal with child behavioral problems; however, when verbal techniques did not help, they reported using physical punishments. These findings demonstrate the perceived importance of child behavioral problems in a rural Nepali community and suggest that multi-level community-based interventions targeting peers, parents, teachers and community leaders could be a feasible and acceptable approach to address the identified problems.
